# Sigmoid colon endometriosis as an uncommon cause of large bowel obstruction: A case report

**DOI:** 10.1016/j.ijscr.2025.111927

**Published:** 2025-09-10

**Authors:** Omar Al Ayoubi, Mohammad Alaa Aldakak, Nizar Alabdullah, Faten Alabdullah, Ayman Alasfar

**Affiliations:** a Faculty of Medicine, Damascus University, Damascus, Syrian Arab Republic; b Al-Assad University Hospital, Faculty of Medicine, Damascus University, Damascus, Syrian Arab Republic

**Keywords:** Endometriosis, Sigmoid colon, Large bowel obstruction, Case report

## Abstract

**Introduction and importance:**

Large bowel obstruction (LBO) is most commonly caused by neoplasms, but rare etiologies like endometriosis should be considered, as bowel involvement can mimic other gastrointestinal disorders and lead to obstruction. Sigmoid endometriosis is a rare but important cause of LBO, which can lead to symptoms ranging from subtle gastrointestinal complaints to overt obstruction. Laparoscopy is the primary diagnostic tool, and surgery is often part of the treatment when obstruction occurs.

**Case presentation:**

We report the case of a 51-year-old Arab female with a history of ulcerative colitis and chronic abdominal symptoms, who presented with progressive distension and intermittent constipation. Colonoscopy revealed a non-passable sigmoid stricture. Surgical resection was performed, and histopathology showed benign endometrial tissue in the colonic wall, confirming sigmoid endometriosis.

**Clinical discussion:**

Endometriosis is a chronic inflammatory condition that can involve various intraperitoneal and extraperitoneal sites, with intestinal involvement reported in up to 37 % of cases—most commonly in the rectum and sigmoid colon. Gastrointestinal symptoms are often nonspecific and may mimic irritable bowel syndrome, making diagnosis challenging. Imaging modalities often lack specificity, while laparoscopy remains the gold standard. In this case, the diagnosis was only confirmed after surgical resection and histopathological analysis. Although medical therapy can be effective in symptom control, surgical excision becomes necessary when obstructive symptoms are present, as seen in our patient.

**Conclusion:**

This case underscores sigmoid colon endometriosis as an uncommon yet significant cause of large bowel obstruction. Awareness of such rare presentations is essential for timely diagnosis and management.

## Highlights

• Sigmoid colon endometriosis is a rare cause of bowel obstruction, often mimicking other GI diseases.
• Symptoms include abdominal distension, constipation, or partial obstruction without classic cyclical signs.
• Imaging and biopsies may be inconclusive; laparoscopy and histology are key for diagnosis.
• Medical treatment helps symptoms, but surgery is needed in obstruction to relieve narrowing.
• Considering endometriosis in colonic strictures aids timely diagnosis and improves outcomes.

## 1 Introduction

Large bowel obstruction (LBO) accounts for approximately 25 % of intestinal occlusions, with neoplasms being the leading cause, responsible for approximately 60 % of cases [1,2]. Other causes include volvulus, chronic diverticular disease, and less commonly Crohn's disease, infections, and endometriosis, which together account for 10–15 % of cases [2,3]. Among the less common causes of LBO, endometriosis is significant, affecting approximately 10–15 % of women of reproductive age [4]. When endometriosis involves the bowel, which is the most common site of extra-pelvic endometriosis, the sigmoid colon and rectum are the predominant locations [5]. It is characterized by the infiltration of endometrial glands and stroma into at least the muscularis propria of the intestinal wall [6]. Clinically, patients with sigmoid colon endometriosis may remain asymptomatic or present with a diverse range of symptoms such as painful bowel movements, abdominal cramps, constipation, diarrhea and vomiting [7]. For establishing a definitive diagnosis, laparoscopic visual assessment of the pelvic cavity remains the gold standard, unless lesions are directly visible in the vagina or at other accessible sites [8]. While medical management is commonly employed to alleviate symptoms and control disease progression, surgical excision becomes essential in cases where medical therapy fails or when significant colonic involvement leads to obstruction or diagnostic uncertainty [9].

In this context, we report the case of a 51-year-old female who presented with partial large bowel obstruction secondary to sigmoid colon endometriosis. The case is clinically significant because the presentation closely resembled other common colorectal pathologies, highlighting the diagnostic challenges and the importance of considering this entity in the differential diagnosis, even in postmenopausal patients.

The work has been reported in line with the SCARE criteria [10].

## 2 Case presentation

A 51-year-old Arab female presented to the gastroenterology outpatient clinic with a three-month history of progressive abdominal distension, intermittent constipation, and lower abdominal discomfort, with cyclical abdominal pain prior to menopause. She denied vomiting or weight loss. Her Gynecological history was unremarkable; she was married with three children, all delivered vaginally. Her Past medical history included ulcerative colitis diagnosed 17 years earlier, and managed medically with 5-aminosalicylic acids (5-ASAs). She had also undergone cholecystectomy around the time of diagnosis. Her regular medications included amiodarone for cardiac arrhythmia and pentazocine for chronic pain. She was a light smoker.

On examination, she was stable with borderline hypotension (90/70 mmHg). Abdominal examination showed distension and diffuse tenderness without guarding or rebound, and bowel sounds were preserved. Given the patient's progressive abdominal distension and intermittent constipation, several differential diagnoses were considered, including inflammatory, neoplastic, and obstructive causes such as colorectal carcinoma and ulcerative colitis stricture. Therefore, colonoscopy was chosen as the initial investigation for direct visualization and tissue sampling, while abdominal X-ray and ultrasound were omitted due to their limited sensitivity in detecting partial large bowel obstruction and mucosal lesions. The procedure revealed a tight, non-passable stricture located approximately 25 cm from the anal verge. Biopsies from the stricture site showed focal chronic inflammation and hyperplastic changes, with no evidence of dysplasia or malignancy.

Given the patient's history of ulcerative colitis and a fixed sigmoid stricture, colorectal malignancy was initially suspected but excluded by biopsies showing only chronic inflammation. A flare of ulcerative colitis was unlikely due to the absence of diffuse mucosal disease, while Crohn's disease was ruled out by the lack of segmental inflammation or granulomas. Other causes of obstruction, such as volvulus or diverticular disease, were excluded by endoscopic and intraoperative findings.

**Fig. 1:**
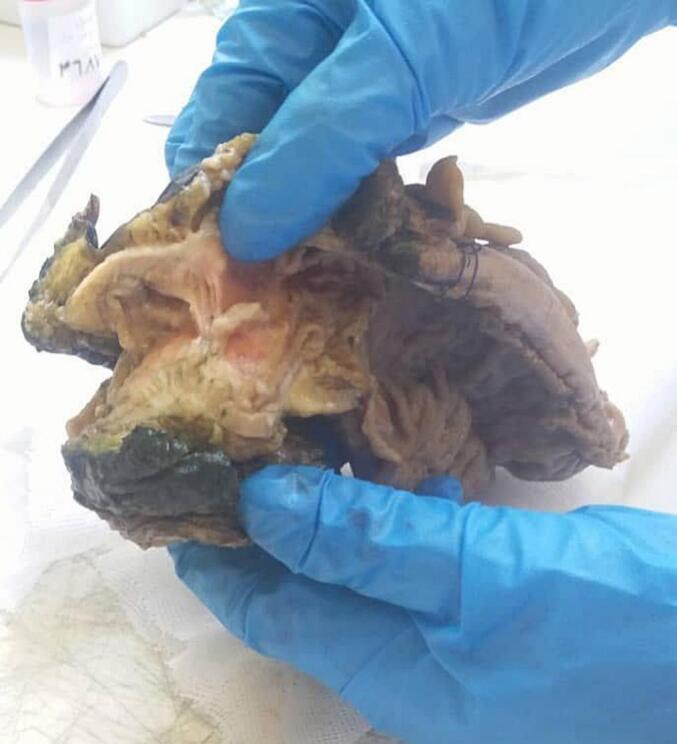
Gross specimen of the resected sigmoid colon showing a firm, whitish, homogenous intramural mass with central mucosal ulceration and wall thickening. The mass measured (5 × 3.5 × 3 cm) and was located (13 cm) from the proximal and (0.5 cm) from the distal surgical margins.

**Fig. 2:**
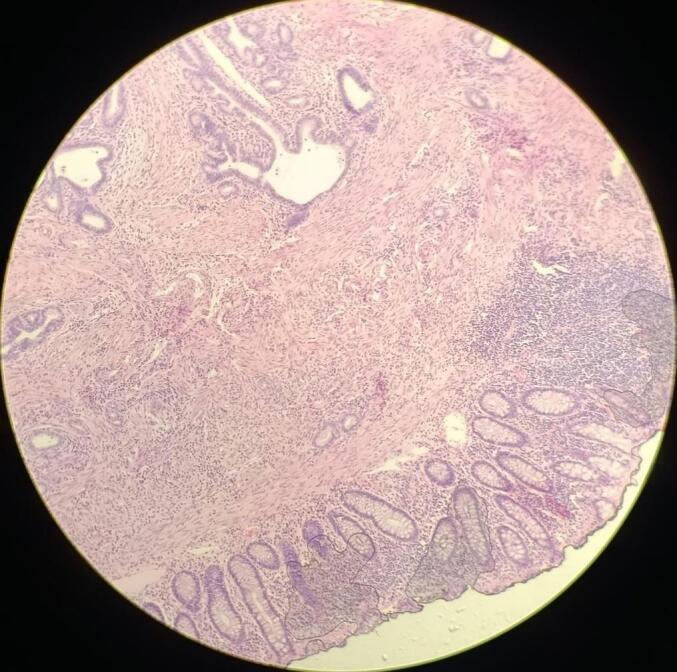
Low-power photomicrograph (H&E stain, ×40) of the sigmoid colon showing distorted colonic architecture with mucosal ulceration, focal cryptitis, and patchy chronic inflammatory infiltrate extending into the submucosa.

**Fig. 3:**
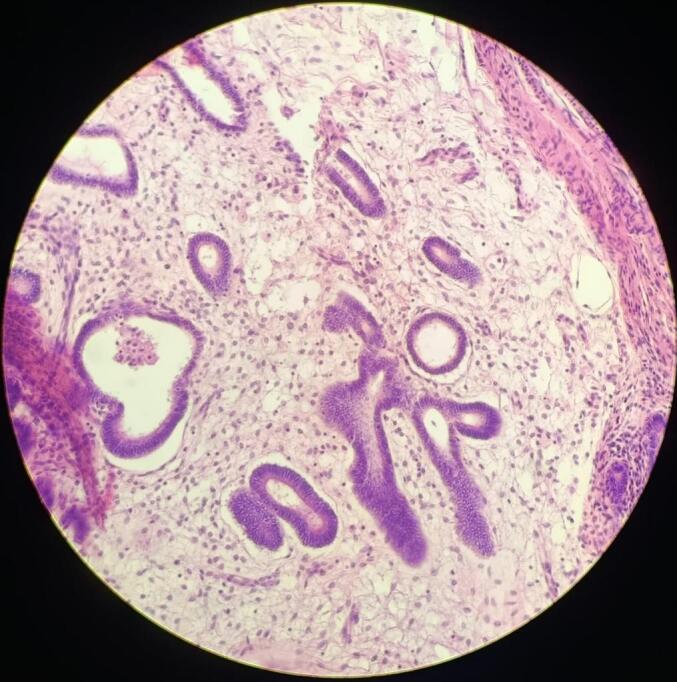
High-power photomicrograph (H&E stain, ×200) revealing benign endometrial glands surrounded by endometrial-type stroma embedded within the muscularis layer of the colonic wall, confirming the diagnosis of intestinal endometriosis.

In light of these findings, the patient was referred for surgery; preoperative workup was normal. An exploratory laparotomy was performed via a midline infraumbilical incision. Intraoperatively, a firm, well-defined mass-like lesion was identified at the site of the sigmoid stricture. A sigmoidectomy was performed with appropriate margins, and the specimen was submitted for histopathological evaluation. Gross examination of the resected sigmoid segment (measuring 18 × 4 cm) revealed a white, homogenous intramural mass (measuring 5 × 3.5 × 3 cm), associated with mucosal erosions **[**Fig. 1**]**. The mass was located 13 cm from the proximal and 0.5 cm from the distal surgical margins, without invasion into the muscularis propria. Microscopic examination revealed benign endometrial glands and stroma within the submucosa, muscularis, and serosa, consistent with sigmoid endometriosis. The colonic mucosa also demonstrated focal active colitis with cryptitis and crypt abscesses [Figs. 2, 3], without evidence of dysplasia or malignancy.

Postoperatively, the patient was counseled about recurrence risk; no hormonal therapy was initiated given her postmenopausal status. She was scheduled for regular follow-up with surveillance colonoscopy. Recovery was uneventful, and at three months she was symptom-free with normal bowel function.

The chronological sequence of events is summarized in [Table 1].

**Table 1:** Timeline of clinical events.

Date/Period	Event	Details
3 months before presentation	Symptom onset	Progressive abdominal distension, intermittent constipation, and lower abdominal discomfort. Cyclical worsening of abdominal pain noted prior to menopause.
Presentation day	Outpatient clinic visit	Physical examination: stable vital signs except borderline hypotension (90/70 mmHg). Abdominal distension and diffuse tenderness without guarding or rebound.
Same week	Endoscopic evaluation	Flexible sigmoidoscopy and colonoscopy revealed a tight, non-passable sigmoid stricture at ~25 cm from anal verge. Biopsies: focal chronic inflammation, no dysplasia or malignancy.
Shortly after endoscopy	Preoperative assessment	Laboratory tests, ECG, echocardiography all within acceptable limits. Patient deemed fit for surgery.
Surgery day	Exploratory laparotomy and sigmoidectomy	Identified firm mass-like lesion at sigmoid stricture site. Resected with margins. Specimen sent for histopathology.
Postoperative period	Recovery	Uneventful recovery, discharged in stable condition.
3 months post-surgery	Follow-up	Patient asymptomatic with normal bowel function. Surveillance colonoscopy planned.

## 3 Discussion

Endometriosis is a chronic inflammatory condition characterized by the presence of endometrial glands and stroma outside the uterine cavity, and was described for the first time in 1690 [11,12]. The disease affects approximately 10–15 % of women of reproductive age, predominantly between 25 and 45 years old [13], and is classified into superficial or peritoneal lesions, ovarian, and deep infiltrating endometriosis (DIE). DIE represents the most severe form, occurring in around 1 % of women of reproductive age and in 14–20 % of those with endometriosis [12,14]. Endometriotic lesions may develop in various locations, including intraperitoneal organs such as the ovaries, uterine surface, fallopian tubes, uterine ligaments, large and small intestines, and appendix, as well as extraperitoneal sites like the inguinal region, vagina, vulva, perineum, lung pleura, skin, muscles, and limbs [15]. Among these sites, intestinal involvement is reported in 3 % to 37 % of all endometriosis cases [16], with the rectum and sigmoid colon being the most frequently affected segments (72 %), followed in decreasing order by the cecal appendix, terminal ileum, cecum, and transverse colon [17]. Patients affected by bowel endometriosis frequently experience a range of gastrointestinal symptoms such as dyschezia, abdominal bloating, constipation, or diarrhea, which may intensify with the menstrual cycle. Additional signs can include the passage of mucus in stools, cyclical rectal bleeding, urgency to defecate, and a sensation of incomplete bowel evacuation [6]. Due to the nonspecific nature of these symptoms, clinical diagnosis is particularly challenging [18], especially given the considerable overlap with irritable bowel syndrome (IBS), which often leads to misdiagnosis as IBS or ‘spastic colon’ in many women [19]. The symptoms observed in our patient, including abdominal distension and intermittent constipation, are consistent with gastrointestinal manifestations described in the literature for bowel endometriosis. In addition, our patient experienced cyclical pain prior to menopause, consistent with the menstrual cycle–related symptom pattern frequently reported in the literature for bowel endometriosis.

Moving on to diagnostic methods, various radiological imaging techniques often fail to reveal specific signs; however, endorectal ultrasound using a radial probe has demonstrated high sensitivity and specificity [20]. More recently, dynamic contrast-enhanced MRI has shown improved diagnostic accuracy [21]. In addition to imaging, histopathological examination serves as another important diagnostic method and requires the presence of both glandular and stromal tissue. Laparoscopy remains the gold standard diagnostic tool, offering an overall sensitivity of 97 %, though its specificity is somewhat lower at approximately 77 %, largely due to its invasive nature [8,22]. In our case, preoperative colonoscopy did not establish the diagnosis, and magnetic resonance imaging was not performed due to resource limitations and logistical constraints. The definitive diagnosis was ultimately made through histopathological examination of the surgically resected specimen, highlighting the particular diagnostic challenge posed by this rare presentation of sigmoid colon endometriosis in our patient.

Regarding treatment approaches, non-steroidal anti-inflammatory drugs (NSAIDs) are useful for relieving endometriosis-related pain. Hormonal therapies that suppress ovarian function for around six months can also reduce symptoms, with options like combined oral contraceptives pills. Depending on disease severity, the ideal approach is to diagnose and surgically remove endometriotic lesions during the same procedure, provided that adequate preoperative consent has been obtained [8]. However, although medical therapy is effective in many patients with endometriosis, our patient ultimately required surgical management due to the presence of a significant mass causing luminal narrowing, which aligns with recommendations favoring surgical excision in cases of obstructive symptoms.

## 4 Conclusion

In conclusion, this case highlights the rare occurrence of endometriosis involving the sigmoid colon causing significant luminal narrowing and partial bowel obstruction. Given the nonspecific clinical presentation and diagnostic challenges, awareness of such atypical manifestations is crucial for timely diagnosis and appropriate management, which may necessitate surgical intervention to prevent serious complications.

## 5 Limitations

This case report is limited by the absence of preoperative imaging, which could have provided additional diagnostic information. Furthermore, as a single case, the findings cannot be generalized, and the diagnosis was only confirmed postoperatively through histopathology.

## Author contribution

**First Author**: **Omar Al Ayoubi**: Conceptualization, data collection, literature review, case analysis, and manuscript writing.

**Second Author**: **Mohammad Alaa Aldakak**: Clinical data interpretation, literature review, and drafting of the discussion section.

**Third Author**: **Nizar Alabdullah**: Manuscript editing assistance, histopathology interpretation, and figure preparation.

**Fourth Author**: **Faten Alabdullah**: Documentation of surgical details, intraoperative image acquisition, and critical manuscript revision.

**Fifth Author**: **Ayman Alasfar**: Reference review, manuscript formatting, final proofreading, and overall project supervision.

All authors have read and approved the final manuscript.

## Ethical approval

Ethical approval for this study (Ethical Committee MD-0910011235-123) was provided by the Biomedical research Ethics Committee BMREC of Damascus University on 1 June 2025.

## Guarantor

The First Author: Omar Al Ayoubi.

## Patient's perspective

“For months, I struggled with bloating, constipation, and discomfort that kept getting worse. I thought it might be related to my previous health issues, but the symptoms were different and worrying. The colonoscopy and surgery were difficult moments for me, but I was relieved to finally have an explanation. After the operation, my symptoms disappeared, and I am now able to live normally again without the discomfort that had affected my daily life.”

## Research registration number

Not applicable.

## Declaration of Generative AI and AI-assisted technologies in the writing process

Artificial intelligence tools (ChatGPT) were used to assist in language editing and improving the clarity of the manuscript. All AI-generated content was reviewed and verified by the authors to ensure accuracy and integrity, and no AI tools were used to generate or fabricate data.

## Funding

This research did not receive any specific grant from funding agencies in the public, commercial, or not-for-profit sectors.

## Patient's consent

Written informed consent was obtained from the patient for publication and any accompanying images. A copy of the written consent is available for review by the Editor-in-Chief of this journal on request.

## Conflict of interest statement

The authors declared no potential conflicts of interest concerning the research, authorship, and/or publication of this article.
